# Nationwide cohort study reveals low bifidobacteria and distinct microbiota composition and function in Swedish newborns

**DOI:** 10.1080/19490976.2026.2719244

**Published:** 2026-08-26

**Authors:** Eleonora Cavani, Alfons Edbom Devall, Yan Chen, Gianfranco Grompone, Nele Brusselaers, Marija Vlajic, Willem M. de Vos

**Affiliations:** a Alba Health AB, Stockholm, Sweden; b BioGaia AB, Stockholm, Sweden; c Department of Women’s and Children’s Health, Karolinska Institute, Stockholm, Sweden; d Global Health Institute, University of Antwerp, Antwerp, Belgium; e Laboratory of Microbiology, Wageningen University, Wageningen, The Netherlands; f Human Microbiome Research Program, University of Helsinki, Helsinki, Finland

**Keywords:** Infant microbiome, metagenome, gut microbiome, bifidobacterium, gut health

## Abstract

In a nationwide study (PREVENT) addressing the longitudinal development of 253 infants in Sweden (744 stool samples), we used deep metagenomic sequencing to determine the bacterial composition. We focused on the 200 fecal samples collected during the first 6 months of life from 146 infants who were predominantly breast-fed. Unsupervised analysis of these infants’ microbial gut composition revealed three distinct foundational community types: CT1 infants had high *Bifidobacterium* spp. levels, CT2 infants had only *B. breve* at low abundances but high *Bacteroidota*, and CT3 infants had virtually no *Bifidobacterium* spp. but elevated levels of pathobionts. Surprisingly, we observed a clear bimodal distribution of *Bifidobacterium* spp., the canonical taxa associated with early-life colonization. Sixty samples from predominantly (58%) vaginally-born infants did not contain any detectable gut *Bifidobacterium* genomes (<0.001%; confirmed by 16S rRNA amplicon sequencing and qPCR analysis). The remaining 140 samples showed high relative *Bifidobacterium* abundance (median 70%), notably including *B. breve, B. longum* subsp. *longum* and *B. longum* subsp. *infantis*. Infants lacking *Bifidobacterium* spp. showed crying after feeding and contained an unusual gut microbiota composition, dominated by known pathobionts such as *Clostridium neonatale* or *Klebsiella michiganensis*. The metagenome-based predicted functional capacity of the gut of infants lacking *Bifidobacterium* spp. showed an increased levels of potentially undesired genes involved in mucus degradation, sporulation processes, and flagella production, while the *Bifidobacterium*-colonized infants were enriched in starch degradation, glutamate transport, and aromatic amino acid production. Notably, we found that a pathway for the production of lactate derivatives of tryptophan and other aromatic amino acids with anti-inflammatory activity was more abundant in the latter group. Our results indicate that infants lacking *Bifidobacterium* spp. contain unusual gut microbiota architecture and functions that are associated with health issues. This pattern is more prevalent in PREVENT than observed in many other studies, underscoring the importance of early testing, while long-term follow-up and potential nutritional advice should be addressed in future studies.

**Clinical Trials:**

NCT06285630.

## Graphical abstract

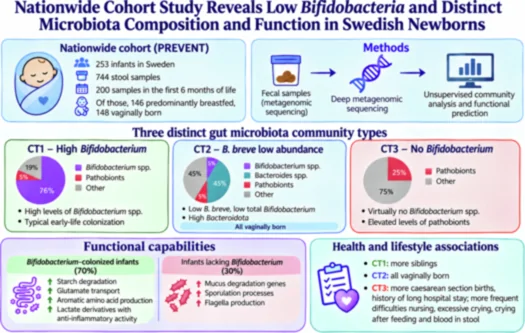

## Introduction

The establishment of the intestinal microbiome in early life and its later maturation play a critical role in shaping human health, influencing immune development, metabolic programming, and protection against pathogens.^1^ Born sterile, the infant’s intestinal tract is colonized rapidly by gut microbes from the mother and to a lesser extent, those from the father.^2-5^ Growing evidence suggests that the early development of the gut microbiome has lasting effects on health later in life, which underscores the importance of understanding the patterns of microbial community assembly.

Recent studies with longitudinal birth cohorts have revealed specific gut developmental community states that are associated with later-life health.^6^ ^,^ ^7^ In the deeply sampled Finnish HELMi cohort of over 1000 infants, the presence of *Bifidobacterium* and *Bacteroides* spp. appeared as early keystone organisms, driving intestinal microbiota development and consistently predicting positive health outcomes, even after 5 y.^6^ Similarly, in a cohort based on the UK Baby Biome Study, including 1288 neonates, *Bifidobacterium* spp., specifically *B. longum* and *B. breve*, dominated community states that were associated with a low abundance of antibiotic resistance genes and long-term pathogen colonization resistance.^7^

While *Bifidobacterium* spp. received particular attention owing to their ability to metabolize maternal milk oligosaccharides (HMOs) and contribute to maintaining a healthy intestinal environment,^8^ emerging studies suggest that the canonical *Bifidobacterium* abundance in the first few months is not universal, even in breast-fed infants. This was already noted last decade in a meta-analysis, where notably US infants showed a very low age-adjusted abundance of *Bifidobacterium* spp.^9^ This low *Bifidobacterium* abundance was partially attributed to technical issues, but with the improved sampling pipelines and metagenome-based analyses, this explanation could be ruled out. Established factors contributing to the absence of *Bifidobacterium* spp. include the use of antibiotics, birth mode and hospital stay or absence of breast-feeding and use of formulas.^9-12^ Metagenomic analysis of newborns in the UK Baby Biome study showed that *Bifidobacterium* spp. were nearly absent in the first week’s gut microbiota in approximately 20% of the infants; instead, their community structure was dominated by the pathobiont *Enterococcus faecalis.* ^7^ Moreover, the My Baby Biome study analyzed the gut microbial composition of infants from 48 of the 50 US states and showed that approximately 25% of those aged 1–3 months lacked *Bifidobacterium* spp.^13^ An early-life microbiota consisting of *Bifidobacterium* spp. with the potential to produce aromatic lactate has been linked to a reduced risk of childhood allergic sensitization.^14^ Hence, the observation that infant guts increasingly harbor reduced levels of *Bifidobacterium* spp. in this foundational gut microbiota has been proposed to be associated with the increased development of non-communicable diseases.^15^

Importantly, not only the mere presence of *Bifidobacterium* spp. seems to be important, but also the species composition. A meta-analysis of 979 stool samples from eight cohorts showed that *B. longum* subsp. *infantis* was abundant in infants from historically high-breastfeeding countries but was almost absent or showed delayed development in low-breastfeeding countries.^16^ Similarly, a comparative meta-analysis of the gut microbiota composition in infants from industrialized and non-industrialized populations revealed a high prevalence of *Bifidobacterium longum* subsp. *infantis* in the Tanzanian population, in contrast to its near absence in some breast-fed infants from high-income countries.^17^ Moreover, a recent survey of over 4000 predicted genomes of *Bifidobacterium* spp. found that *B. longum* subsp. *infantis,* capable of degrading HMOs and plant glycans, dominated early-life microbiota in low-income but not high-income countries, suggesting new avenues for developing tailored bifidobacterial supplements.^18^

To explore the situation in continental Europe, we investigated the gut microbiota in 744 fecal samples of 253 Swedish infants in the nation-wide PREVENT Study during their first 2 y of life using deep metagenomic sequencing. A recent review documented the gaps in early-life microbiome research and identified the need for longitudinal studies in unselected cohorts.^19^ Specifically, Sweden is a relevant country for birth cohorts since follow-up and extensive data collection are feasible because of the general science interest and its national registry, while its population has become considerably heterogeneous in recent decades. Focusing on the 200 fecal metagenomes of infants sampled in the first 6 months of life to provide a coherent group where most metadata have been collected, we confirmed the presence of three foundational distinct community types by unsupervised clustering, differing markedly in their *Bifidobacterium* genomic content, microbial composition, and associated health outcomes. This led us to examine a central question: whether *Bifidobacterium* spp. absence in the first 6 months, increasingly observed in breast-fed infants from high-income countries, indeed extends to continental European populations, what microbial communities and functional genes are established in its place, what temporal stability these have, and whether these genes are associated with health and other metadata. Understanding the prevalence of distinct early-life community types, their microbial composition, and their associations with factors such as delivery mode and health outcomes is critical for evaluating the conventional assumption of ubiquitous *Bifidobacterium* colonization.

## Materials and methods

### Study population and sampling

The PREVENT Study is a nationwide longitudinal study monitoring the microbiome composition of generally healthy newborn infants in Sweden. The study was approved by the Swedish Ethical Review Authority (Etikprövningsmyndigheten, Case No. 2023-05299-01). Participants were recruited using a multi-faceted strategy, with families registering their interest via a dedicated website landing page on Alba Health AB’s web domain. Recruitment initiatives included both local outreach and social media campaigns. The social media strategy comprised paid advertisements on Meta, targeting people located in Sweden, between ages 25 and 38, organic posts on LinkedIn, Instagram, Reddit, and parental Facebook groups, as well as a paid collaboration with the Swedish women-only social media community “Heja Livet.” Other recruitment involved the local distribution of flyers at private hospitals and children healthcare centers in Stockholm, along with posters at Umeå University. The inclusion criteria for the PREVENT study included that the infant was generally healthy, term-born, resident in Sweden, and between 1 and 365 d old at the initial sampling timepoint. The exclusion criteria included major health problems, preterm birth, or long-term use of antibiotics or proton-pump inhibitors. The study used a longitudinal design based on the date of initial enrollment, not fixed infant ages. Of the 627 families who registered interest, 253 met the inclusion criteria. The participants provided three fecal samples: the initial sample (*n* = 253), a second sample collected approximately 3 months later (*n* = 248), and a third sample collected approximately 6 months after the initial sample (*n* = 243), resulting in a total of 744 samples. The interquartile ranges of ages at the different timepoints were 131–264 d, 217–348 d, and 328–467 d, respectively. A total of 241 families provided all three longitudinal samples. Written informed consent was obtained from the legal guardian of the enrolled infants. For each fecal sample, parents completed questionnaires to provide additional information at each timepoint (see Supplementary Table S1). This included household environment details (including housing, number of occupants, and pets), family health information (including parent and sibling health status, parental mental health), and child-specific data (including medication, sleep/crying, gastrointestinal health, diagnosed conditions, and stool appearance). Additionally, peripartum factors were captured, encompassing antibiotic use, delivery mode, and conditions, such as preeclampsia and gestational diabetes. Finally, dietary information for the infants was collected, including feeding practices and the use of supplements.

### Fecal sample collection

Three fecal samples were collected from each PREVENT participant, at approximately 3-month intervals starting in February 2024. The parents were instructed to take a photograph of the stool sample before scraping it from the diaper using the provided spoon. The samples were collected using DNA/RNA Shield™ Fecal Collection tubes from Zymo Research, Germany, which have been shown to stabilize fecal samples and their DNA.^20^ The parents were instructed to firmly close the tube lid and shake the tube vigorously in an up-and-down motion for at least 30 s. Sampling and logistics were conducted at room temperature until processing. The sealed tubes were placed in plastic bags and mailed in pre-stamped envelopes directly to Zymo Research, Germany, where they were stored at 4 °C until further processing within weeks. The samples were destroyed 3 months after DNA extraction.

### DNA extraction and metagenomic sequencing

Genomic DNA was extracted from infant fecal samples using the ZymoBIOMIC®-96 MagBead DNA Kit following the manufacturer’s protocol. The metagenomic libraries were constructed using the Illumina DNA Library Prep Kit (Illumina, San Diego, CA, USA) using unique dual-index 10 bp barcodes with Nextera® adapters (Illumina, Sandiego, CA, USA). Genomic DNA was then prepared for whole-genome sequencing with 2 × 300 nucleotide paired-end chemistry on the Illumina NovaSeq X Plus platforms, generating, on average 15 M paired-end reads (total 30 M reads) per sample. For each DNA extraction and sequencing run, parallel positive and negative controls were included. The negative control was a blank sample, and a sample containing the ZymoBIOMICS® Microbial Community Standard (Zymo Research Corporation) was used as a positive control.

### Metagenomic data processing and taxonomic profiling

Metagenomic data were processed using the StaG metagenomic workflow collaboration, v0.7.0.^21^ The pipeline integrates a series of quality control and taxonomic classification tools optimized for host-associated microbiome datasets. First, fastp v0.23.2^22^ was applied to remove low-quality bases, adapters, and short reads based on default parameters. To remove host-derived sequences, filtered reads were aligned against the Genome Reference Consortium Human Reference 38 (GRCh38) using Kraken2, v2.1.2.^23^ Reads identified as of human origin were discarded, and only non-host reads were retained for taxonomic profiling. Samples with fewer than 15 million reads after host sequence removal were excluded from further analysis. The remaining samples had a mean read count of 32.9 million (IQR: 27.0–36.8 million reads). The taxonomic composition of the microbiota was analyzed using MetaPhlAn, v4.0.6,^24^ which relies on clade-specific marker genes to provide species-level resolution. Profiling was performed using the marker gene database version mpa_vOct22_CHOCOPhlAnSGB_202212. To improve the quantification specificity of *B. longum* subspecies, the database was manually augmented by the inclusion of two representative genomes: *B. longum* subsp. *infantis* and *B. longum* subsp. *longum*.^25^ Functional annotation was obtained using HUMAnN^26^ version 3.7, using chocophlan v296.201901 for the nucleotide search and the full Uniref90 database for the translated search.

### Community clustering

The bacterial community types were clustered at the genus level using the partitioning around medoids (PAM) algorithm, implemented via the “pam” function in the R package cluster.^27^ This method was applied to dissimilarity matrices generated with the vegdist function from the R package “vegan”.^28^ Four distance metrics were evaluated: Bray‒Curtis dissimilarity, Pearson distance, robust Aitchison distance, and a standard Aitchison distance. For the latter, a pseudocount equal to one-tenth of the minimum non-zero relative abundance was added to the data prior to the centered log-ratio transformation. Fisher's exact test was used to evaluate the associations between all categorical metadata variables and cluster membership. A *p*-value of less than 0.05 was considered statistically significant; adjustments for multiple testing were done using the Benjamini‒Hochberg method and are mentioned where appropriate.

### Functional analysis

The HUMAnN utility function humann_regroup_table was used to obtain KEGG Orthologous groups, then names were added with humann_rename_table, and finally, the counts were normalized to relative abundance using humann_renorm_table. Similarly, the MetaCyc pathways were obtained from HUMAnN. To identify differentially abundant KOs, MaAsLin2^29^ was used with standard, univariate-style testing based on the relative abundance of the gut *Bifidobacterium* spp. using the “LOG” transformation parameter. All associations were corrected for false discovery rate (FDR).

### Metagenomic pipeline confirmation by use of an alternative database, 16S rRNA gene amplicon sequencing, and qPCR

To verify and corroborate the metagenomic pipeline, fecal samples from an infant with no detectable levels of gut Bifidobacterium spp. were selected, and DNA was extracted as described above and analyzed by metagenomic sequencing using an alternative for the StaG pipeline, 16S rRNA gene amplicon sequencing, and qPCR.

To corroborate the routinely used StaG pipeline, an alternative shotgun metagenomic database, Web of Life (WoL) release 2.0,^30^ was used. Initially, fastq files, which had undergone quality control and host removal via the StaG pipeline, were aligned to the WoL database using bowtie2.^31^ Subsequently, woltka^32^ was employed to generate taxonomic tables. This was achieved using the woltka classify command with parameters set for phylum, genus, and species ranks, as well as coords and maps, against the WoL release 2.0 taxonomy database.

For 16S rRNA gene amplicon sequencing, the V3‒V4 regions were amplified using the Quick-16S™ Primer Set V3‒V4 from Zymo Research. Paired-end sequencing (2 × 300 bp) was conducted on an Illumina NextSeq platform. To generate the taxonomic tables, the dada2 pipeline^33^ was used, using the recommended settings in the official documentation (https://benjjneb.github.io/dada2/tutorial.html). The database used was Greengenes2 (toGenus, version 2024_09), provided by the maintainers of the dada2 package.^34^

Species-specific qPCR assays were employed for precise and sensitive quantification of *Bifidobacterium* spp. and *R. gnavus* populations that were the most abundant in the infant DNA samples. The reactions, totaling 24  μL per reaction, utilized PowerTrack SYBR Green Master Mix (Applied Biosystems), which includes SYBR Green dye for fluorescence-based detection of double-stranded DNA, along with the appropriate primer sets. The signals were corrected for average 16S copy number according to rrnDB,^35^ using copy numbers of 6, 5, and 4 for the total bacterial count and *Bifidobacterium* spp., respectively. For estimating the total bacterial count, the forward and reverse primers were AACGCGAAGAACCTTAC and CGGTGTGTACAAGACCC, respectively.^36^ The forward and reverse primers used for *R. gnavus* were TCCGGATTTACTGGGTGTAAAG and CTCTAGCCTGACAGTTCCAAAT,^37^, and for *Bifidobacterium* spp. TCGCGTCYGGTGTGAAAG and CCACATCCAGCRTCCAC, respectively.^38^

### Statistical analysis

Statistical analysis was conducted using R (version 4.2.2) and Python (version 3.12). Data visualizations in R were created with ggplot2,^39^ while matplotlib^40^ was used for Python visualizations. The Python package Plotly^41^ was utilized for the Sankey visualization. The geographic distribution of the participants was visualized as a heatmap using Datawrapper. All the statistical tests considered a *p*-value of 0.05 as the threshold for significance.

### Data availability

The metagenome sequences in this study have been deposited in the European Nucleotide Archive (ENA) under accession code PRJEB100868.

## Results

**Figure 1.**
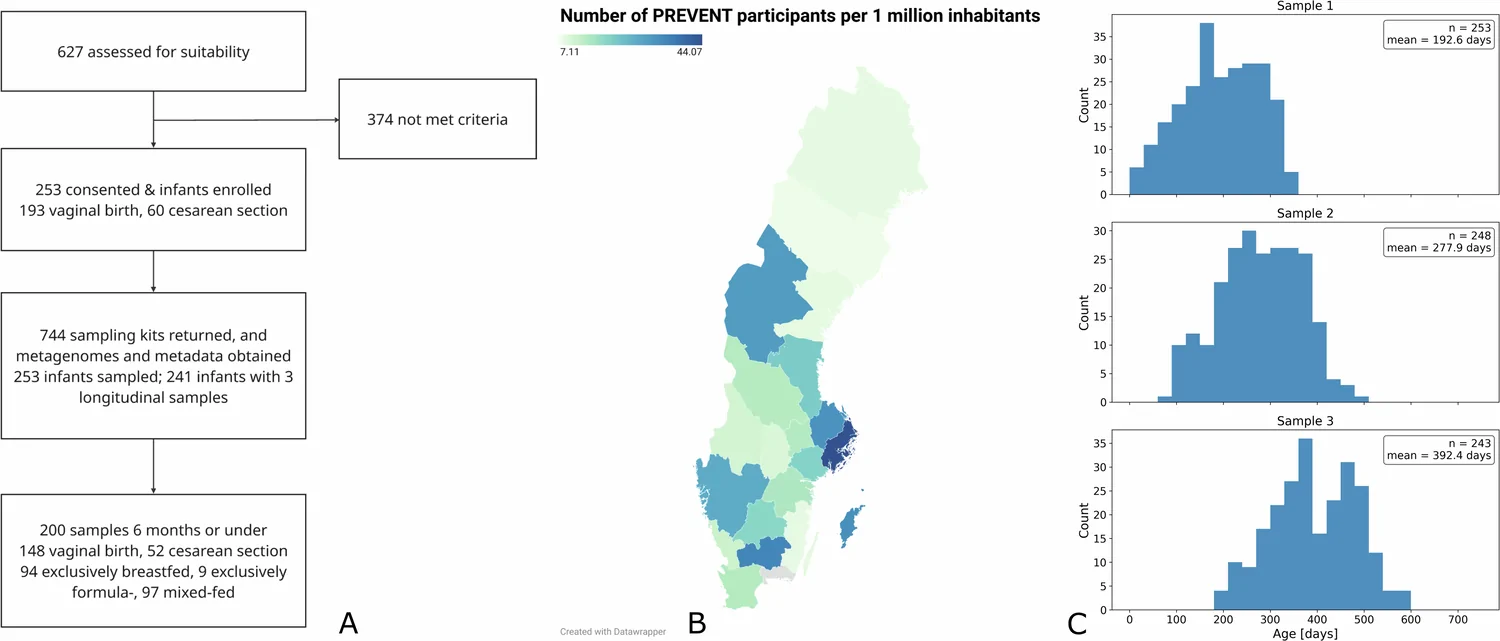
PREVENT cohort and samples used in this study. Flow diagram of trial recruitment and key data (left), geographic distribution of recruited families across Swedish counties based on postal codes shown as participants per million inhabitants (middle), and the age distribution of all 744 PREVENT fecal samples collected, shown by sampling timepoint (samples 1, 2, and 3 collected approximately 3 months apart; right).

The PREVENT Study (NCT06285630) recruited families across Sweden with term-born newborns (up to 12 months) using social media outreach to ensure as broad as possible geographic representation. Within 4 weeks, over 600 families responded, of whom 253 qualified, consented to participate in the study, and provided the first infant stool sample (Figure 1). The families were asked to provide 3 stool samples at approximately 3-month intervals, along with corresponding infant metadata, resulting in 744 samples in total (253, 248, and 243 at each time point, respectively). A total of 241 families provided all 3 longitudinal samples, and the age of the infants at the time of sampling ranged from 12 to 593 d (Figure 1C). While even distribution of sampling is difficult to obtain and postal code data of the PREVENT participants showed some concentration in the capital area and large cities (combined population of approximately 8 million), we also observed major representation of participants from the less densely populated rural counties (approximately 2.5 million inhabitants) (Figure 1B; see also Supplementary Figure S1). Since we excluded preterm infants, the gestational age ranged from 32 to 37 weeks (median 35). The frequency of cesarean section delivery in the entire cohort was 26%, which is slightly higher than the national average (13%–21% depending on the region).^42^

### Gut microbiota community types and associations with health

**Figure 2.**
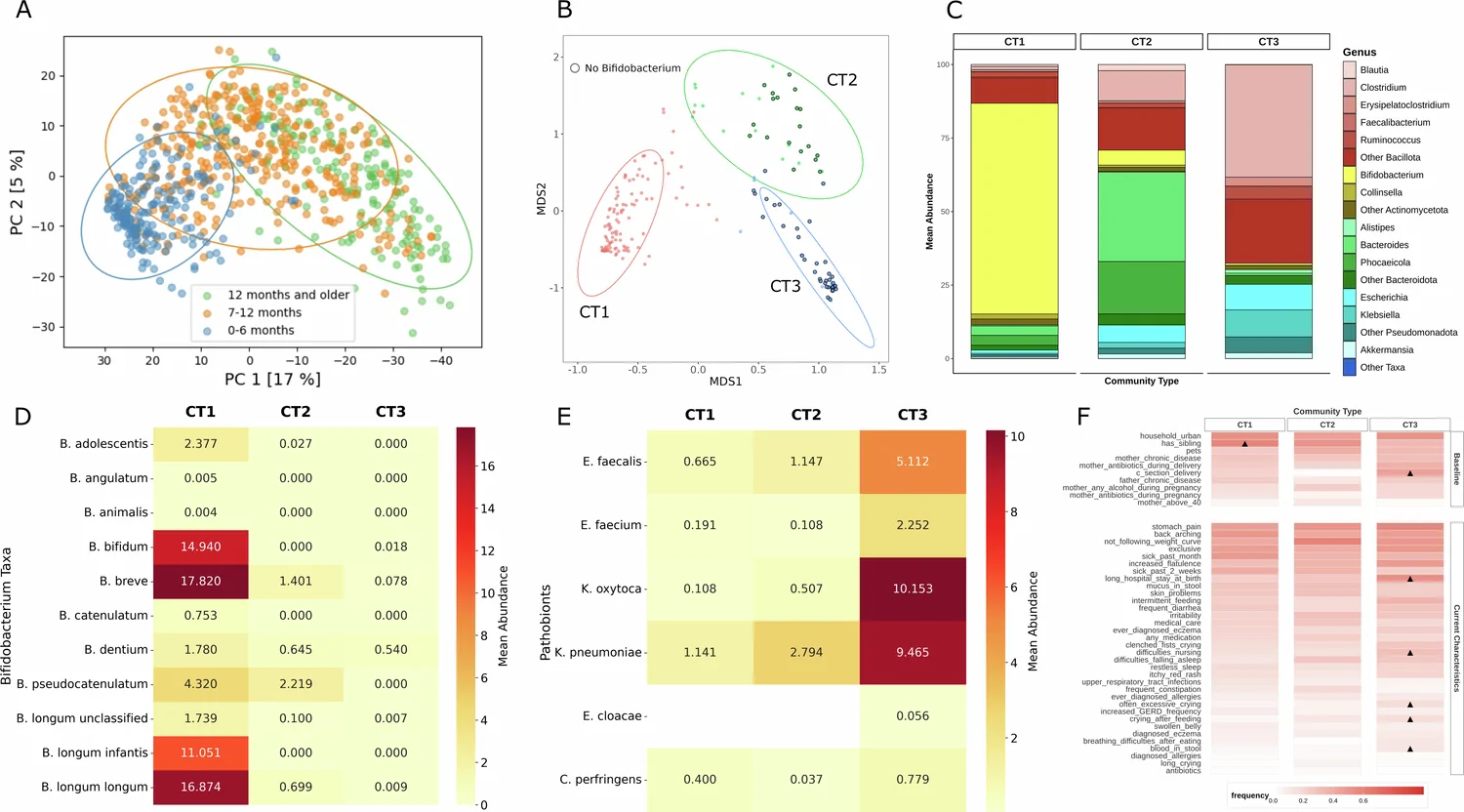
Infant gut microbiota analysis and foundational community type clustering with associated health and other characteristics. (A) Principle Coordinate Analysis (PCoA) of the genus-level microbiota showing all 744 of the 253 recruited PREVENT infants. Age groups are indicated by color (blue 0–6, orange 7–12, and green over 12 months). (B) PCoA of the genus-level microbiota of the 200 samples of the 0–6 month old PREVENT infants showing 3 CTs. Samples without detectable *Bifidobacterium* spp. are indicated by circles. (C) Genus-level relative abundance by CTs. (D) Heatmap of *Bifidobacterium* species abundance across CTs. (E) Heatmap of pathobiont relative abundances across CTs. (F) Heatmap of parent-reported metadata (see Supplementary Table S1) grouped into baseline data (top) and characteristics (bottom) of infants belonging to the three CTs. The triangle indicates *p* < 0.05. After multiple testing, cesarean section (q = 0.012, log2 odds ratio = 1.96) and long hospital stay at birth (q = 0.013, log2 odds ratio = 1.92) remained significant.

DNA extracted from 744 fecal samples of the recruited infants (Figure 1) was subjected to deep metagenomic sequencing and subsequent bioinformatic analysis. Principal coordinate analysis (PCoA) of gut microbiota data showed separation by infant age, with a gradient from the first 6 months (left) to over 12 months (right) (Figure 2A). We next focused on the infant samples (*n* = 200) collected during the first 6 months of life, as it is in this period that the use of mothers’ milk is almost universal (Figure 1), most metadata were obtained, and the foundation is provided for important physiological, immune, and metabolic developments.^43^ These samples were derived from a total of 146 infants with a mean age of 4.5 months, as 54 infants provided two samples (see below and Supplementary Figure S2). The gut metagenomes of these infants were subjected to unsupervised genus-level clustering to identify the foundational community types (CTs)^15^ (Figure 1). Multiple distance metrics tested showed consistent segmentation into 3 clusters, and Bray‒Curtis dissimilarity clustering revealed 3 community types (CT1, CT2 and CT3) characterized by distinct microbial compositions (Figure 2B and 2C). The gut microbiota samples of infants in CT1 (*n* = 114) were dominated by *Bifidobacterium* spp. (median abundance 76%, IQR [60%–89%]) and had relatively low amounts of *Bacteroides* spp., while samples in CT2 (*n* = 34) had low levels of *Bifidobacterium* spp. (~5%) and high levels (~45%) of *Bacteroides* spp. in addition to *Clostridium* spp. CT3 (*n* = 52) contained samples of infants with almost no *Bifidobacterium* spp., dominated by *Clostridium* spp., and including a high level of *Klebsiella* spp. (~10%). The background data indicated similar breastfeeding patterns, maternal age, and probiotic use rates, but showed exclusive vaginal delivery in the CT2 infants. The latter infants also reported the lowest usage levels of prepartum antibiotics. The highest frequency of cesarean section delivery was found in the CT3 infants (Table 1). It has been reported that the use of probiotic supplements, notably those containing *Bifidobacterium* spp., may be a driver of gut microbiome development in early life.^5^ ,^44^ When addressing the use of probiotics, we noticed a trend toward the highest use in infants belonging to CT3 (Table 1).

**Table 1.** Background data and metadata.

Relevant background data	CT1	CT2	CT3
**Male/Female**	56/58	16/18	30/22
**No siblings/With siblings**	48/66	17/17	34/18
**Cesarean section/Vaginal**	26/88	0/34	26/26
**Exclusive/Partial breastfed**	54/60	14/20	26/25***
**Mean maternal age (years)**	33.9	35.4	33.9
**Probiotics (Yes/No)**	46/68	10/24	23/28*
**Antibiotics prepartum (Yes/No)**	16/98	2/32	9/43
**Antibiotics intrapartum (Yes/No)**	29/79**	7/27	19/28**

Relevant background data	Undetected		Detected
**Cesarean section/Vaginal**	**25/35**		27/113
**Exclusive/Partial breastfed**	31/28***		63/77
**Male/Female**	33/27		69/71
**No siblings/With siblings**	36/24		63/77
**Mean maternal age (years)**	34.0		34.3
**Probiotics/No probiotics**	24/35*		55/85
**Antibiotics prepartum (Yes/No)**	8/52		19/121
**Antibiotics intrapartum (Yes/No)**	20/35**		35/99**

**Figure 3.**
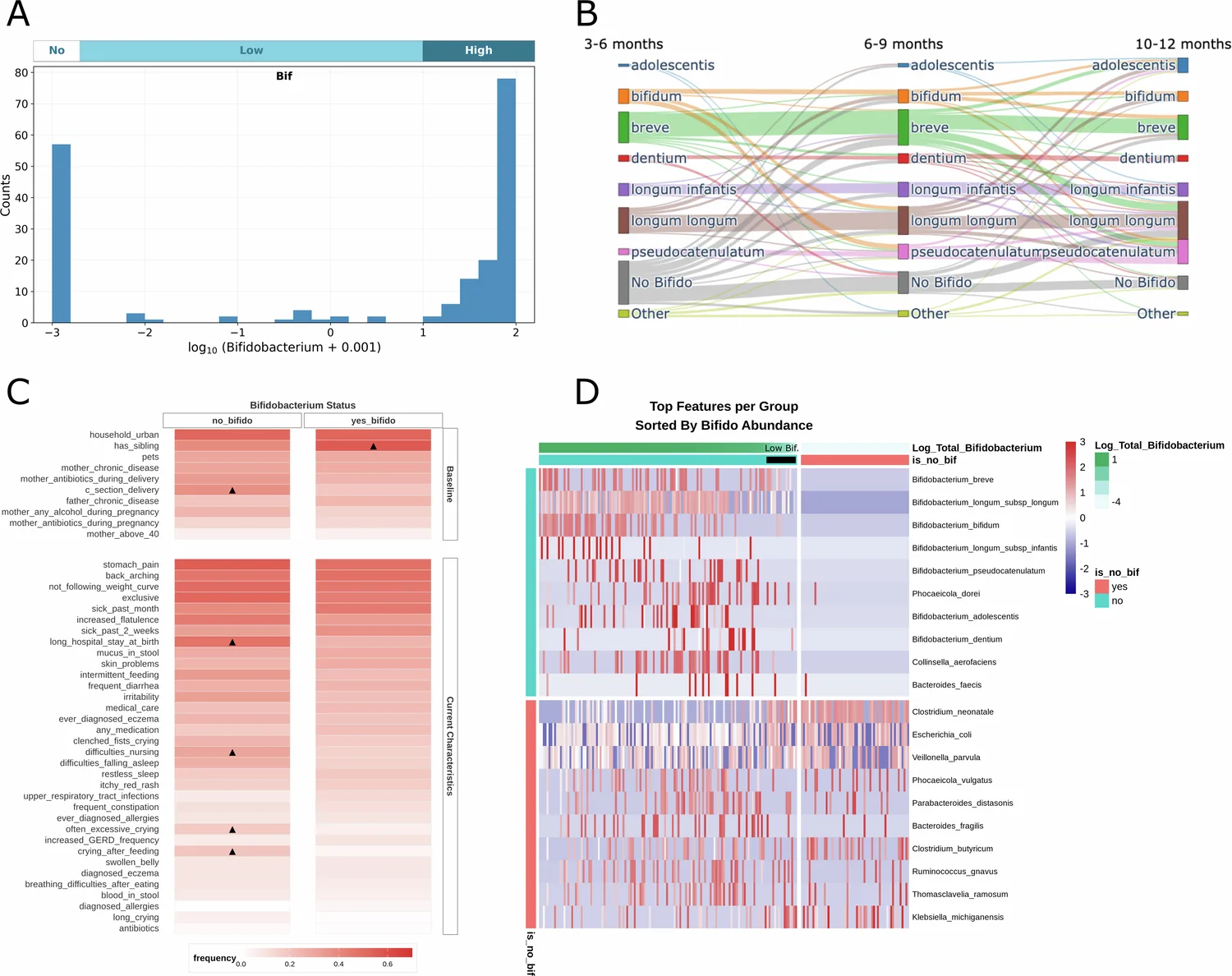
*Bifidobacterium* distribution in PREVENT infants in the first 6 months of age with stability, health associations, and major composition. (A) Distribution of the relative abundance of *Bifidobacterium* spp. in the 200 samples from 146 PREVENT infants during the first 6 months, plotted on a log scale, showing a characteristic bimodality. No-Bifidobacterium samples have levels below detection (0.001%). Low *Bifidobacterium* samples are indicated (over 0.001% but below 10%). (B) Development of *Bifidobacterium* spp. over time. (C) Heatmap of parent-reported metadata (see Supplementary Table S1) grouped into baseline data (top) and characteristics (bottom) of infants belonging to the three high-CT categories. The triangle indicates *p* < 0.05. After correcting for multiple testing, long hospital stays at birth (0.034, log2 odds ratio = 1.47) and crying after feeding (q = 0.025, log2 odds ratio = 2.66) remained significant. (D) Heatmap of log scale levels of the top 10 species in samples containing *Bifidobacterium* spp. and no-*Bifidobacterium* samples. The low *Bifidobacterium* samples are indicated by the black bar.

Relevant background data of the mothers and their infants that provided fecal samples in the first 6 months of life, as well as relevant metadata of the infants. These bacteria were grouped based on their gut microbiota into the three Community Types, CT1 (*n* = 114), CT2 (*n* = 34), and CT3 (*n* = 52) (top) or into the ones where detectable levels of *Bifidobacterium* spp. were absent (n=60) or present (*n* = 140) (bottom). Significant differences are indicated in bold (see also Figures 2F and 3C).

^*^ For one sample, we do not receive information on the probiotic use.

^**^ Missing samples responded with "I don't know" on the intrapartum question.

^***^ There is one sample for which we don't have feeding data.

The distribution of *Bifidobacterium* spp. in the CTs (Figure 2D) showed that the gut microbiota of infants belonging to CT1, and to a lesser extent CT2, contained the early colonizing species *B. breve*, *B. longum* subsp. *longum* or *B. longum* subsp. *infantis*; in the gut of CT2 infants, mainly *B. pseudocatenulatum* and *B. breve* were found. Consistent with their high levels of *Klebsiella* spp., the CT3 infants had also elevated other gut pathobionts (>25%), notably *E. faecalis* and *E. faecium*. In the infants belonging to CT1 and CT2, the gut pathobionts levels were relatively low (~5%; Figure 2E).

The clustering of samples was stable over time in these first 6 months: of the 54 infants that provided two samples in this period, 39 (72%) remained in the same cluster (Supplementary Fig. S3). Health and other metadata provided by the families were analyzed across the 3 CTs (Figure 2F and Table 1). CT1 infants more frequently report having siblings compared to the other CTs. CT2 infants showed no significant correlations to any health data, but were all vaginally born, whereas CT3 infants were delivered more often via cesarean section and showed a history of long hospital stay – while not significant after multiple testing, these infants also tended to experience difficulties in nursing, often excessive crying, crying after feeding, and blood in the stool (Figure 2F). One possibility is that the potential difficulties in nursing may have reduced the interactions with the infant, limiting the transfer of *Bifidobacterium* spp. Both maternal and paternal transmission of *Bifidobacterium* spp., including *B. longum*, have been demonstrated previously.^2^

### Absence of gut colonization by *Bifidobacterium* spp. in a significant fraction of infants

Given the reports of reduced levels of *Bifidobacterium* spp. in the US and UK and the potential association with long-term health effects,^15^ we addressed their genomic abundance and associations with metadata in the Swedish PREVENT cohort. Analysis of the relative abundance revealed a characteristic bimodal distribution of *Bifidobacterium* spp. (Figure 3A). Among the 200 infant samples, 60 (30%) showed *Bifidobacterium* spp. below the detection level of 0.001% (Figure 3A). These samples (referred to hereafter as no-*Bifidobacterium* samples) were mainly grouped into clusters CT2 and CT3 (see Figure 2B). In contrast, the remaining 140 samples were found to contain readily detectable levels of *Bifidobacterium* spp., with two dozen samples with low (0.001%–3%) levels and over 100 samples of very high relative abundance levels of the gut *Bifidobacterium* spp. (between 10% and 90%, with a median of 70%) (see Figure 3A). All of these genes were predominantly assigned to cluster CT1. The dominant species in these infants’ guts included *B. breve* (21%), *B. longum* subsp. *longum* (16%) or *B. longum* subsp. *infantis* (9%), and *B. bifidum* (11%) (Figure 3A).

Since the fecal samples of the PREVENT infants were collected longitudinally at 3-month intervals covering a period of up to 2 y (see Figure 1C), the distribution of the different *Bifidobacterium* species (and *B. longum* subspecies) could also be followed over time by analyzing all 744 analyzed samples (Figure 3B). Interestingly, the colonization patterns of *Bifidobacterium* spp. remained highly stable in this period; this held especially true for *B. longum* subsp. *infantis* and *B. breve*. Moreover, approximately half of the infants who had no gut *Bifidobacterium* spp. in the first 6 months of life stayed devoid of these early colonizers throughout most of their first year.

To confirm that the unusually high fraction of no-*Bifidobacterium* infants was not an analytical artifact, we corroborated the metagenomic pipeline based on the high-throughput Illumina NovaSeq chemistry by characterizing fecal DNA from a 3-month-old infant with no detectable levels of *Bifidobacterium* spp. This included repeated metagenomic sequencing, computational analysis using an alternative to the StaG pipeline, 16S rRNA gene amplicon sequencing, and bifidobacterial-specific qPCR (see Supplementary Figure S3). The taxonomic profiles deduced from the original and re-sequenced data, using both the primary MetaPhlAn pipeline and an alternative Web of Life (WoL) database, were highly identical (Spearman correlation coefficients of 0.999 and 0.997, respectively). Moreover, the phylogenetic composition of samples without detectable *Bifidobacterium* spp. and high levels (approximately 40% relative abundance) of *R. gnavus* was confirmed with 16S rRNA amplicon sequencing (Fig. S4). Finally, by using qPCR analysis, we confirmed the absence of *Bifidobacterium* spp. (<0.00003%) and quantified *R. gnavus* to over 10^9^ cells per gram of feces, corresponding to ~35% relative abundance when corrected for 16S rRNA copy number. The inability to detect *Bifidobacterium* spp by metagenomics was confirmed by analyzing another infant fecal sample, where qPCR showed the relative abundance to be approximately 0.00002%. Hence, the results obtained with these two infant samples corroborated the metagenomic pipeline and indicated that the unusual gut microbiome composition of the no-*Bifidobacterium* samples was not due to sequencing or pipeline errors.

The 60 no-*Bifidobacterium* samples were found to derive from infants that were more frequently delivered by cesarean section than the 140 samples that contained these canonical gut colonizers (42% vs 19%) (Table 1). Other relevant background and metadata did not differ significantly between the groups apart from the fact that siblings were more frequently reported for infants with *Bifidobacterium*-containing samples. This also holds for the breastfeeding practices that did not differ between the two groups: approximately half of the infants were exclusively breastfed, the other half received mixed feeding, and only 4% were exclusively formula-fed (see Figure 1; Table 1). It has been reported that the use of probiotic supplements, notably those containing *Bifidobacterium* spp., can drive gut microbiome development in early life.^5^, ^44^ However, the reported probiotic use was highly similar between the groups (Table 1). Moreover, *Lactobacillus*-based probiotics were used regularly, but we noticed only sparse use of *Bifidobacterium*-containing supplements – hence we consider interference probiotics with the *Bifidobacterium*-based classification to be unlikely.

Geographic analysis showed that the no-*Bifidobacterium* infants were twice as common in the less densely populated rural county of Jämtlands län compared to the urban areas such as Stockholm or Gothenburg (45% vs. 20% of participating families; *p* = 0.002; see Supplementary Figure S1). We did not study this further, as we have no exposure, dietary intake, or socioeconomic data for the families in this region.

**Figure 4.**
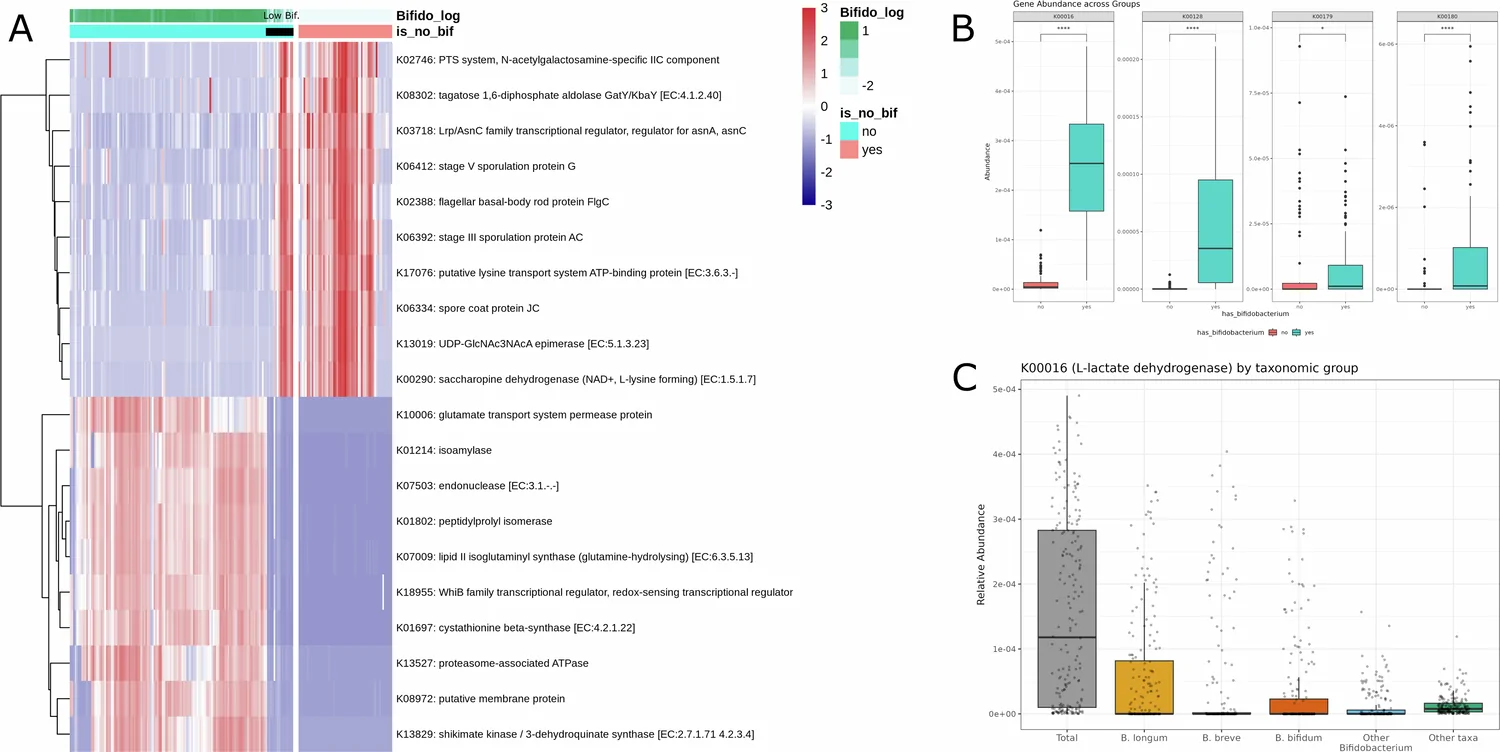
Metagenome-based predicted functions in relation to *Bifidobacterium* levels and distribution of K00016 among *Bifidobacterium* species. The KEGG-predicted functions of no-*Bifidobacterium* samples were compared with those samples containing detectable levels of *Bifidobacterium* (Supplementary Table S4). (A) Heatmap of the top 10 predicted functions in the two groups, with the low *Bifidobacterium* samples indicated by the black bar. The KEGG gene numbers are indicated. (B) Distribution of genes involved in the production of lactate derivatives of aromatic amino acids, including the aromatic lactate dehydrogenase (K00016) as well as the aldehyde dehydrogenase K00128 and the alpha and beta subunits of indolepyruvate ferredoxin oxidoreductase (K00179 and K00180) – see text. (C) Species distribution of the predicted aromatic lactate dehydrogenase K00016 in the dataset of samples containing *Bifidobacterium* spp.

Stratification by the relative abundance of *Bifidobacterium* spp. showed that all the negative health correlations were linked to the no-*Bifidobacterium* infants (Figure 4C). Compared to those that were colonized by detectable levels of *Bifidobacterium* spp., these no-*Bifidobacterium* infants significantly more often had a history of long hospital stays and showed more crying after feeding. As expected, the symptoms such as difficulties during nursing and excessive crying (though not significant after multiple testing) aligned very well with those of the infants belonging to CT3 that include most no-*Bifidobacterium* samples (see Figure 2D).

### Microbiota composition of infants with and without Bifidobacteria spp.

The remarkable dichotomic presence of *Bifidobacterium s*pp. in the early-life gut of the PREVENT infants was subsequently studied in detail, and samples with differential abundance of *Bifidobacterium* spp. were analyzed for their taxonomic composition, focusing on species prevalence, their predicted function, and their ability to utilize HMOs, since virtually all infants had received mothers’ milk. The top ten prevalent species in the infant samples with *Bifidobacterium* spp. and those in the no-*Bifidobacterium* samples were found to differ significantly (Figure 3D; Supplementary Table S2). Notably, the samples with low levels of *Bifidobacterium* spp. showed a composition very much resembling that of the no*-Bifidobacterium* samples, suggesting that a certain level of *Bifidobacterium* colonization was needed to provide colonization resistance to the non-canonical species (Figure 3D black bar).

The ten most abundant species that abundantly colonized the infants with *Bifidobacterium* spp. mainly encompassed those that are known to utilize HMOs for growth, either completely or partially. These included *Bifidobacterium* spp. (with *B. breve, B. longum*, and *B. bifidum* as the most prevalent and complete HMO degraders), as well as *Phocaeicola dorei* (previously known as *Bacteroides dorei*), *Collinsella aerofaciens*, and *Bacteroides faecis* , which degrade only a subset of the known HMOs. As there are indications that infants in Western countries are losing *B. longum* subsp. *infantis* strains that have a full repertoire of HMO-utilizing genes,^18^ we also addressed the presence of this subspecies in comparison with that of *B. longum* subsp. *longum*. In line with the findings in the US and UK, Swedish PREVENT infants were also abundantly colonized by *B. longum* subsp. *longum* but only sparsely by *B. longum* subsp. *infantis*; close inspection suggested that the latter subspecies was notably present when the *B. bifidum* was not detectable (Figure 3D). Remarkably, the samples of all no-*Bifidobacterium* infants contained virtually none of these HMO-degrading species, and only one sample with *Phocaeicola dorei* and one with *Bacteroides faecis* were detected (Figure 3D).

High relative gut abundances were observed for the set of top ten species that were consistently prevalent in the no-*Bifidobacterium* infants (Figure 3D and Supplementary Table S1). The highest average prevalence (73%) and relative abundance (24%) were observed for *Clostridium neonatale*, a potential opportunistic pathogen linked to necrotizing enterocolitis in preterm infants.^45^ *C. neonatale* was also detected in *Bifidobacterium*-positive infants, though at a lower prevalence, and was most frequent among those with low rather than high *Bifidobacterium* levels. Following in prevalence (above 41%–67%) were the well-known early-life Gram-negative colonizers *Escherichia coli*, *Veillonella parvula*, and several *Bacteroidia (Bacteroides fragilis*, *Phocaeicola vulgatus*, and *Parabacteroides distasonis*). A recent study showed that virtually all newborn infants are colonized by maternal *E. coli*, but receive protective antibodies from their mothers’ milk – this early *E. coli* colonization may evoke an early-life immune response in the newborn that may protect for later infections of pathogenic strains.^46^ This may explain the abundance of *E. coli* colonization in the infants with *Bifidobacterium* spp. Normal colonization also includes the maternal transfer of *V. parvula*, which produces propionate from lactate, a metabolic end-product of early-life anaerobic fermentations. This potential pathobiont that colonizes the oral cavity and upper intestinal tract was also found in some samples from infants with high levels of *Bifidobacterum* spp. The detected members of *Bacteroidia* that were also prevalent in the infants that contain *Bifidobacterium* spp., also constitute a normal part of the maternally transferred microbiota and are capable of utilizing HMOs, which will promote their persistence.^47^ No HMO utilization has been described for *Clostridium butyricum* ^48^ that was more prevalent in no-*Bifidobacterium* infants as compared to those with detectable *Bifidobacterium* spp. In contrast, *Ruminococcus gnavus* (also known as *Mediterraneibacter gnavus*) is known to contain the enzymatic machinery to degrade HMOs and has been described as a gut symbiont and pathobiont.^49^, ^50^ It is remarkable that *R. gnavus* was also found to be prevalent in infants with high gut levels of *Bifidobacterium* spp. This also holds true for *Thomasclavelia ramosa*, also termed *Erysipelatoclostridium ramosum* and formerly known as *C. ramosum*, a common butyrate producer in the gut of infants after weaning and in adults.^51^ Quite prevalent and abundant bacterium in the no-*Bifidobacterium* samples was *Klebsiella michiganensis*, which is much less abundant in infants with high levels of gut *Bifidobacterium* spp. In recent years, *K. michagenensis* has been described as an emerging hospital-acquired bacterial pathogen that often contains antibiotic resistance genes and has been implicated in neonatal sepsis.^52^

Several species have appeared sporadically but in extremely high abundances (25%–79% relative abundance) in the gut microbiomes of infants without *Bifidobacterium* spp. (Supplementary Table S2). Some of these genes may be associated with health, such as *Akkermansia muciniphila*, which can grow on HMOs and is known to increase barrier function, potentially protecting against pathogen infections.^53^ Other genes are linked to hospital sites (*Enterobacter roggenkampii, Citrobacter koseri, Streptococcus vestibularis, Enterocloster clostridioformis, Actinomyces urogenitalis, Blautia faecis*, and *Sellimonas intestinalis*), associated with eczema in early life (*Hungatella hathewayi*) or are not well-characterized, such as *Collinsella* SGB47280.

### Metagenome-based function prediction of infants with or without detectable *Bifidobacterium* spp.

To understand the predicted functions in the early-life PREVENT infants, we analyzed the metagenome-predicted Kyoto Encyclopedia of Genes and Genomes (KEGG) pathways genes that differed between the intestinal tracts of infants either with or without *Bifidobacterium* spp.

We first analyzed the MetaCyc pathway classifications that revealed significant differences in differential abundances in close to 300 pathways (Supplementary Table S3). The most discriminating (approximately 100-fold differential abundance) pathway in the gut samples of infants containing *Bidifobacterium* spp. was the Bifidobacterium shunt, also known as the phosphoketolase pathway, which is unique for these bacteria and explains the efficient production of acetate and lactate.^54^ Other prominent pathways (over 4-fold differential abundance) included fatty acid biosynthesis, vitamin B1 (thiamine) production, as well as the degradation of pyrimidine ribonucleosides that are abundant in mothers’ milk and starch degradation capacity, which are likely linked to supplementary starchy diets. The MetaCyc-predicted pathways in the infants without *Bifidobacterium* spp. were rather heterogeneous, with only up to 10-fold differential abundance that was observed for the phytol degradation pathway, suggesting a link with a plant-based diet.

Subsequently, we focused on differentially abundant genes that revealed a large set of significantly discriminating functions that varied in up to several thousand-fold in abundance (Supplementary Table S4). An unsupervised heatmap of the top ten discriminating functions present in the infants with or without *Bifidobacterium* spp. showed a clear separation (Figure 4A). An exception was found in the samples that did contain *Bifidobacterium* spp. but to a level below 1%, and these were grouped with the no-*Bifidobacterium* samples (indicated by the black bar), testifying for the unbiased assignment.

The functional capacity of the infants carrying no or low levels of intestinal *Bifidobacterium* spp. were enriched in functions involved in special sugar degradation pathways, such as transport of *N*-acetylglucosamine (GlcNac) (likely derived from mucus degradation), tagatose 1,6 diphosphate cleavage (a key function of lactose degradation via the lactose PTS pathway), and UDP-GlcNAc-epimerase (likely involved in GalNac degradation, also derived from mucus), as well as enzymes involved in lysine production and transport system as well as an Lrp-like regulator usually involved in amino acid metabolism. A remarkable enrichment in spore-associated genes was detected, including spore coat protein JC, and stage III and stage V sporulation proteins, indicative of the fact that the bacteria colonizing the infants devoid of *Bifidobacterium* spp. are mainly spore-formers, also termed sporobiota that may colonize infants.^55^ A similar enrichment in the FigC protein involved in flagella biosynthesis was observed in the no-*Bifidobacterium* samples, which is of interest in view of the special capacity of bacterial flagelins to signal to the TLR5 receptor and induce a local or systemic inflammatory response, affecting cardiometabolic or gut health.^56–58^ In contrast, the infants that did contain high levels of intestinal *Bifodobacterium* spp. were enriched in other unique metagenome-predicted functions (Supplementary Table S4). These included functions related to adaptation to the oxygenated early-life gut, such as the *Actinobacterium*-specific WhiB family of stress response regulators containing Fe-S clusters,^59^ the cystathionine beta-synthase potentially involved in redox homeostasis, and the proteasome-associated ATP-ase and peptidylprolyl-isomerase, which are involved in the folding of intracellular and secreted proteins. Moreover, various enzymes involved in housekeeping functions were enriched, including the GatD enzyme involved in peptidoglycan biosynthesis, a putative phage holin and the NucS endonuclease involved in mismatch repair. Importantly, metabolic enzymes such as isoamylase and glutamate permease were highly discriminative and likely reflect adaptations to starch-rich diets at weaning and the high level of glutamate in breast milk.^60^

A highly noteworthy enzyme was shikimate kinase, which is involved in the biosynthesis of aromatic amino acids, such as tryptophan. While some *Bifidobacterium* spp. are capable of synthesizing tryptophan,^61^ some use the breastmilk-derived sources and can convert this into indole-lactate (ILA) and indole-acetate (IAA), which have important signaling functions in health and diseases.^62^ Hence, we next studied the pathway genes involved in the biosynthesis of these tryptophan derivatives and discovered that key enzymes in the pathways leading to IAA and ILA from indole-pruvate were enriched in the metagenomes of infants containing gut *Bifidobacterium* spp. (Figure 4B). These included the alpha and beta subunits of indolepyruvate ferredoxin oxidoreductase (K00179 and K00180) as well as the aldehyde dehydrogenase K00128, which are directly involved via indolacetaldehyde in IAA production and were highly and significantly increased in the samples containing *Bifidobacterium* spp. (Figure 4B; Supplementary Table S4). Recently, it has been discovered that the production of ILA from indol-pruvate involves a specialized *B. longum* aromatic lactate dehydrogenase.^63^ This aromatic lactate dehydrogenase (BLLJ_RS05690) is annotated by KEGG as K00016 and is over 20-fold more enriched in the samples containing *Bifidobacterium* spp. (see Figure 4B; Supplementary Table S3). When we analyzed the origin of K00016 in these samples, we found it to be mainly derived from *B. longum, B. breve*, and *B. bifidum* (see Figure 4C). These three *Bifidobacterium* spp. are known to contain this specific aromatic lactate dehydrogenase and have recently been shown to produce a variety of signaling molecules, including ILA from tryptophan, phenyllactate (PLA) from phenylalanine, and hydroxyphenyllactate (4-OH-PLA) from tyrosine.^14^

## Discussion

Since the development of molecular approaches to study microbial succession in neonates several decades ago^64^, a great variety of longitudinal birth cohort studies have addressed early-life gut microbiome assembly, focusing on bacteria, fungi, and their viruses, and their relations with the host.^6^, ^7^ ^,^ ^17^ ^,^ ^65-67^ These cohorts mainly derived from recruitments at specialized medical centers, regional birth clinics, or selected sociodemographic regions, making them and their results inherently biased towards either urban or rural populations. Here, we report on a nationwide recruitment of newborns in the PREVENT study in Sweden, where families of generally healthy infants (up to 1 y of age) were enrolled via social media and analyzed for the gut bacterial assemblies in early life by deep metagenomic sequencing. Within weeks, this resulted in the enrollment of 241 families that provided three consecutive fecal samples (in total, 744) from their newborns, linked to electronic health, family history, and lifestyle records at 3-month intervals. The geographic distribution of the recruited families showed some hot spots in urban regions but also illustrated the effectiveness of social media-based recruitment and remote sample collection, since all counties of Sweden were covered, including rural regions that are relatively sparsely populated (see Figure 1). The frequency of cesarean section birth in the PREVENT cohort (26%) was higher than the Swedish average of 19% and may reflect the interest of the parents in participating, although regional differences cannot be ruled out.^42^

In this study, we focused on the PREVENT infants that were sampled in the first 6 months of life, and our findings either challenged or confirmed earlier observations. A notion that is challenging the canonical view of *Bifidobacterium* ubiquity in early life is our finding that 60 of the 200 samples from infants living in urban areas, and relatively more often in areas, lacked completely *Bifidobacterium* spp. (below the detection level of 0.001%), despite continued breastfeeding in the first 6 months of their life, and mostly (58%) being born vaginally. Supporting recent studies was our finding that the infants could be grouped based on their gut metagenomes into three foundational community types (CTs) with unique signatures that showed differential associations with health.^6^ ^,^ ^7^ ^,^ ^13^ Similar to previous studies, we found that infants belonging to CT3 had a unique microbiota signature with the lowest level of *Bifidobacterium* spp. and relatively high levels of pathobionts that showed an association with health issues, including crying and blood in the stool.

It has been reported that birth by cesarean section is more frequently associated with the absence of the gut *Bifidobacterium* spp. than vaginal birth, which is likely due to compromised mother–infant transmission.^68^ Cesarean section was significantly more frequent among no*-Bifidobacterium* infants than among those with detectable *Bifidobacterium* spp. (42% vs. 19%, see Table 1). Yet a significant fraction (58%) of no-*Bifidobacterium* infants were vaginally born, indicating that delivery mode alone cannot account for the absence of gut *Bifidobacterium* spp. The same holds for antibiotic and probiotic use (see Table 1), indicating that other factors are likely to contribute, such as prolonged hospital stay after birth, compromising maternal transfer or the absence of *Bifidobacterium* spp. in the maternal seeding. Notably, approximately half of the infants who lacked gut *Bifidobacterium* spp. stayed devoid of them for another 6 months (Figure 2). This may also reflect the absence of siblings, as these are known to contribute to *Bifidobacterium* colonization.^69^ The possibility that our findings were based on technical artifacts was ruled out, and we corroborated our approach across multiple pipelines, 16S rRNA amplicon sequencing, and qPCR with fecal infant DNA, confirming that we have robust methodologies (Supplementary Figure S3). The observed bimodal distribution of *Bifidobacterium* spp. in the PREVENT cohort is indicative of the existence of tipping points related to alternative stable states observed earlier in the gut microbiota of adults (Figure 2).^70^ Recently, a conceptual framework for these stable states, termed the ecological network balance index, was proposed based on temporal microbiota development.^71^ It would be of interest to apply this to longitudinal sampling of the infant microbiota and study the involvement of the bimodal distribution of *Bifidobacterium* spp. levels in the transitions to alternative stable states. High rates of *Bifidobacterium* spp. absence has recently been reported in infants aged 3 months or younger in the UK Baby Biome and US My Baby Biome studies (20% and 25%, respectively^7^ ^,^ ^13^). However, the prevalence in PREVENT infant samples was even higher (30%), and the absence of *Bifidobacterium* spp. was shown to persist for 6 or more months in half of the infants, including an important window of early-life development (Figure 3B).

Various studies have indicated that the presence of *Bifidobacterium* spp. in early life improves the immune response^72^ and vaccine efficiency.^73^ Recent association and mechanistic studies have attributed this finding specifically to the *Bifidobacterium*-dependent production of aromatic amino acid-derived compounds (ILA, PLA, and 4-OH-PLA) that are known to interact with the aryl hydrocarbon receptor AhR and induce anti-inflammatory responses and a host of other protective functions.^63^ The presence of the aromatic lactate dehydrogenase-containing *B. longum, B. breve*, and *B. bifidum* that we detected abundantly in the *Bifidobacterium*-containing PREVENT infants (Figure 4C), have been reported to be key in the production of these signaling metabolites (notably 4-OH-PLA) and shown to lower the risk of allergenic sensitization in early life.^14^ We did not find a significant association of these *Bifidobacterium*-containing infants with reduced allergy-related symptoms (Figure 3C), but this can be attributed due to a limited follow-up period.

Detailed analysis of infants devoid of *Bifidobacterium* spp. identified a variety of highly abundant species (see Figure 3D and Supplementary Table S2). These included other common early-life colonizers, such as *Bacteroides* spp. and related genera that can degrade HMOs, but also several unusual, remarkable, or special species. The most abundant and highest-prevalence species was surprisingly *C. neonatale,* a potential opportunistic pathogen associated with necrotizing enterocolitis in preterm infants.^45^ A genomic survey of *C. neonatale* strains showed considerable variability but could not pinpoint specific genes involved in the presumed pathogenicity.^74^ A recent study indicated that the *C. neonatale* virulence could be associated with its metabolic potential since a strain that produced only acetate from lactose was incapable of infecting a gnotobiotic NEC model.^75^ All PREVENT infants were reportedly term-born, and while some suffered from slightly compromised health (Figure 2), no serious events or hospital visits were recorded. Another highly abundant species was *Thomasclavelia ramosa,* also termed *Erysipelatoclostridium ramosum* and formerly known as *C.* r*amosum,* which is often found in the gut of young infants and persists in adult life.^51^ In the MAGIC meta-analysis of infant gut metagenomes, *T. ramosa* was found to be correlated with childhood allergy.^76^ However, studies on the relation of *T. ramosa* (*E. ramosum*) with breastfeeding, it was established that human milk IgA dampened its undesired Th17 immune response, and this is of interest as all PREVENT infants were breastfed.^77^

A special case is the high abundance and prevalence of *R. gnavus* (also known as *Mediterraneibacter gnavus*) that was found in all the infant samples up to 6 months, but in the highest numbers in the infants devoid of *Bifidobacterium* spp. (Figure 3D). *R. gnavus* is a well-known gut inhabitant that has been known for decades to be present in the early-life gut.^64^ ^,^ ^78^ However, blooms of mucus-located *R. gnavus* have been associated with inflammatory bowel disease and may involve the selfish sialidase it encodes.^79-81^ In line with our findings of increased crying in the no-*Bifidobacterium* infants is the observation in the Dutch KOALA birth cohort that members of the *R.gnavus* group are associated with more colicky symptoms below 6 months, although the used 16S rRNA sequencing approach did not allow for specific species assignments as in our metagenome-based analysis.^82^ Moreover, in a recent study of colonization programs in the CHILD cohort, *R.gnavus* was identified as a late colonizer and linked to asthma development.^83^ *R.gnavus* and the earlier mentioned *Clostridium*-like spp. are known to produce resistant spores, explaining not only the metagenome-predicted abundance of sporulation genes (Figure 4A) but also a possible route of dissemination.^55^

A key validating finding was the identification of three community types by unsupervised clustering of the gut metagenomes from 200 PREVENT infants under half a year of age (Figure 2). CT1 was dominated by *Bifidobacterium* spp. (~70% average relative abundance), including *B. longum*, *B. breve*, *B. bifidum*, and *B. infantis* (17%, 15%, 14%, and 12% relative abundance, respectively). These infants were generally healthy, despite low levels of *K. pneumoniae* (~1%), suggesting that the properties of the microbial community could compensate for potential negative effects.^84^ CT2 was characterized by moderate levels of *Bifidobacterium* spp. (almost exclusively *B. breve*), relatively high *Bacteroides* and *Phocaeicola* spp., and was associated with increased irritability and constipation. CT3 had virtually no *Bifidobacterium* spp. and high levels (up to 25% relative abundance) of *Klebsiella* and *Enterococcus* spp., which could explain the observed associations with slightly compromised health, including excessive crying, crying after feeding, and blood in the stool. The presence of these pathobionts in CT3 infants could be rationalized by their more frequent cesarean section delivery and history of long stays in hospitals where *Klebsiella* and *Enterococcus* spp. often originate from.^85^ In recent years, community clusters with similar numbers and general composition were discovered in cohorts in Finland, UK, and US.^6^ ^,^ ^7^ ^,^ ^13^ Infants in clusters with high *Bifidobacterium* spp. were generally healthier than those in clusters with the lowest level, a finding we also observe here in the PREVENT cohort. In the Finnish HELMI cohort, infants with low *Bifidobacterium* spp. had increased risks of allergy, atopic diseases, and asthma^6^ at the 5-year follow-up. The 2-year follow-up in the US My Baby Biome showed that infants with *B. breve* (but not *B. longum)* had a significantly reduced relative risk of adverse immunological outcomes.^13^ Infants in the PREVENT cohort belonging to CT1 also had a high abundance of *B. breve* (22%), but we did not observe specific negative correlations with allergy or atopy, potentially due to the short follow-up period of 1 y (Figure 1). In addition, we did not study the HMO type that has recently been found to correlate with the development of eczema, a primary manifestation of atopy.^66^

This study has several strengths and limitations. The deep metagenomic analysis of early-life samples generated new insight into the composition and predicted functionality of the colonizing microbiota. The nationwide recruitment via mainly social media provided regional insight in the microbiota development of newborns. This revealed high rates of non-canonical microbial colonization patterns that appeared to be most abundant in rural areas. Earlier surveys in Sweden and neighboring Nordic countries recruited participants via hospitals or medical centers and showed different trajectories.^6^ ^,^ ^86^ Limitations include only 1 y follow-up, absence of health registry or socioeconomic status data, and our focus on high-throughput metagenomic characterization with sampling systems that precluded metabolomics analyses. In addition, we did not analyze the mothers’ microbiota, the HMO composition of the breast milk, or the infants’ metabolome, which could support the predicted differential production of signaling aromatic lactic acids in the infants with variable levels of *Bifidobacterium* spp. Moreover, no specific interventions were advised for infants who are colonized early on with unusual or undesired microbes. However, many microbial supplements have been tested for their efficacy to advance health in newborns.^87^ ^,^ ^88^ It is likely that these can be used in conjunction with the early diagnostics to provide science-based nutritional and other recommendations for shaping the early-life microbiome and promoting a healthy development.^19^

## Supplementary Material

FINAL REVISION Cavani et al Supplementary Materials.pdf FINAL REVISION Cavani et al Supplementary Materials.pdf

Cavani et al KGMI Supplementary Table S4.xlsx Cavani et al KGMI Supplementary Table S4.xlsx

Cavani et al Supplementary Table S1.xlsx Cavani et al Supplementary Table S1.xlsx

Cavani et al KGMI Supplementary Table S3.xlsx Cavani et al KGMI Supplementary Table S3.xlsx
