# Supplementary material for: Nationwide cohort study reveals low bifidobacteria and distinct microbiota composition and function in Swedish newborns
Source: Gut Microbes. 2026 Aug 26;18(1):2719244. doi: 10.1080/19490976.2026.2719244 (PMC13523984; doi:10.1080/19490976.2026.2719244)
Supplement: Supplementary file 1 [file KGMI_A_2719244_SM5296.pdf]

Supplementary Materials

Supplementary Fig. S1. Geographic distribution of PREVENT infants devoid of detectable gut *Bifidibacterium* spp. Colors show the counties where infants providing samples with no-*Bifidobacterium* spp. in the first 6 months are located (presented as percentage of the PREVENT infants).

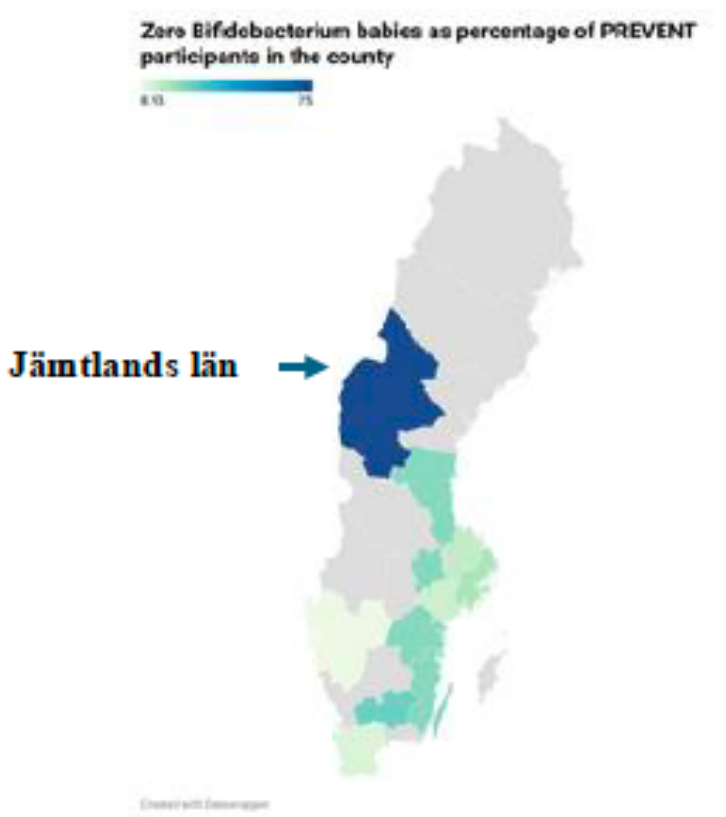

Fig. S2. Age distribution of infants that provided the 200 gut samples in the first 6 months of their life. Their median age was 4.5 months.

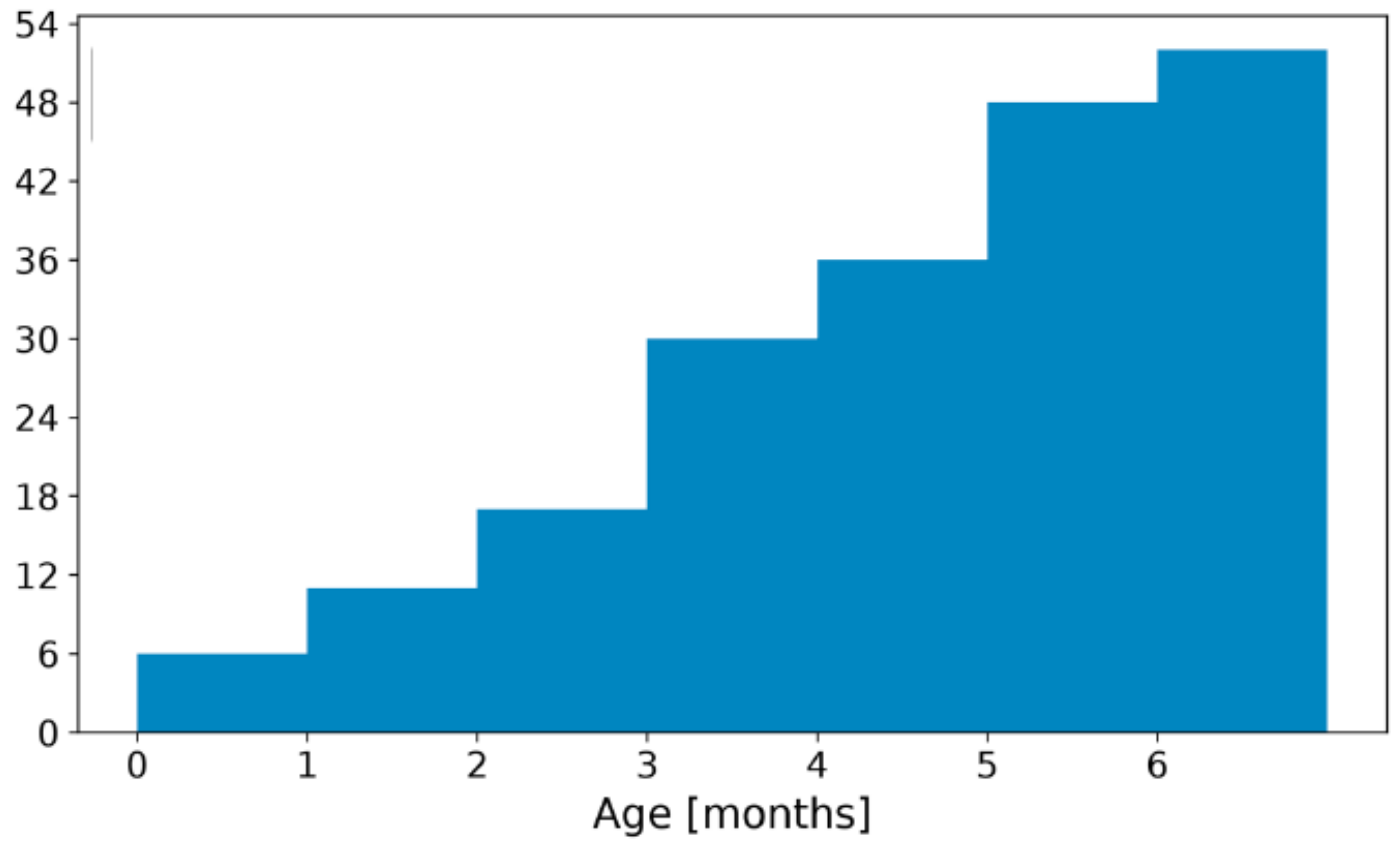

Fig. S3. Temporal stability of samples belonging to the different community types. A total of 54 infants provided two samples in the first 6 months (Phase 1 and 3 months later Phase 2 – see Fig. 1C). A total of 39 (72 %) remained in the same cluster (see diagonal) with those in cluster 1 being the most stable.

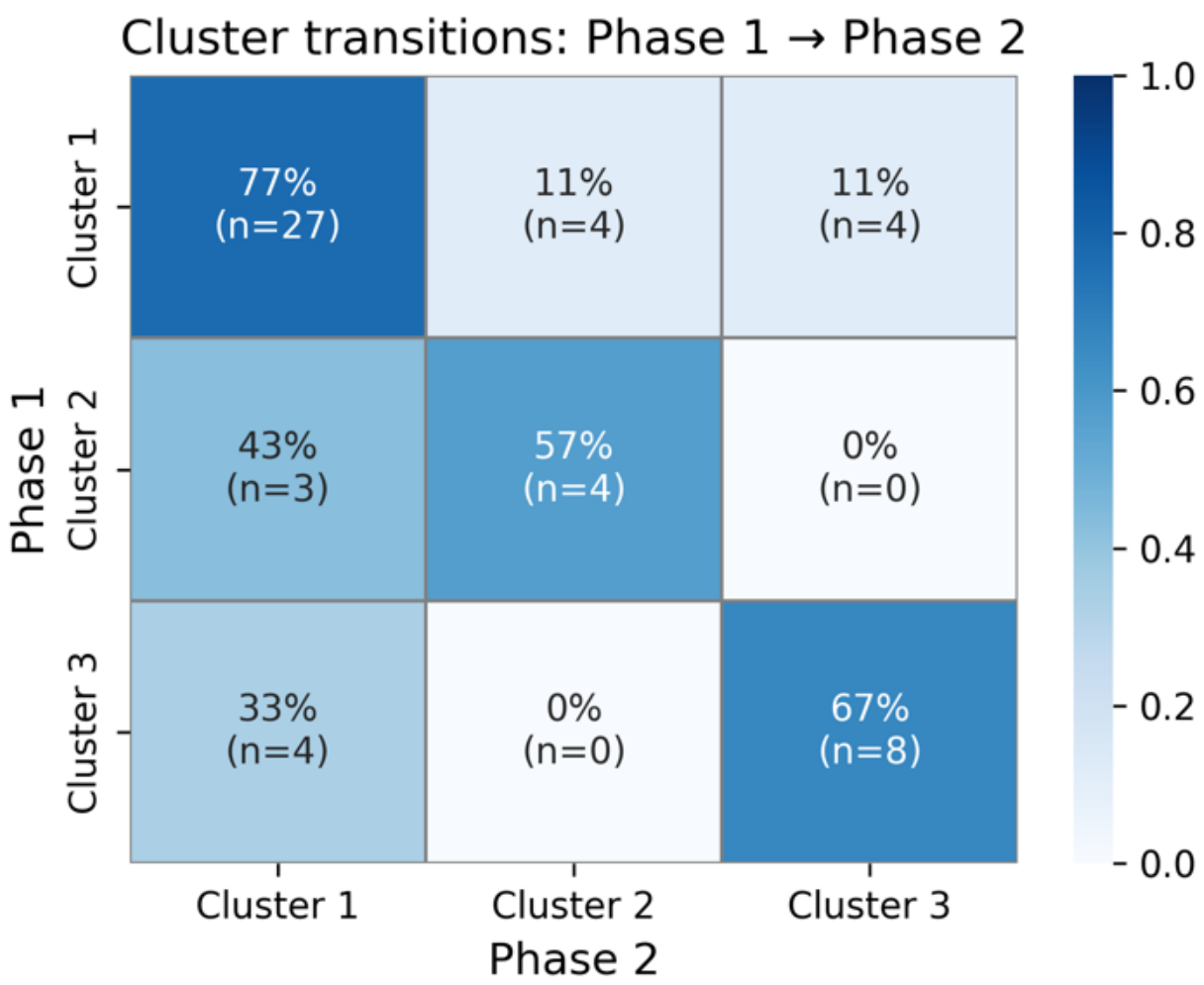

Fig. S4. Reproducibility and corroberation of the metagenome based pipeline. Species composition of gut samples of an infant devoid of *Bifidobacterium* spp. at 3 months of age based on metagenome analysis using Metaphlan (left), (middle) (both two independently sequenced samples demonstrating reproducibility) and on 16S rRNA amplicon sequencing analysis using the Greengenes database (right). The high relative abundances of *Ruminococcus* (40-50 %), *Veillonella* (20-25%) and *Enterococcus* (10-15 %) is evident in all analyses.

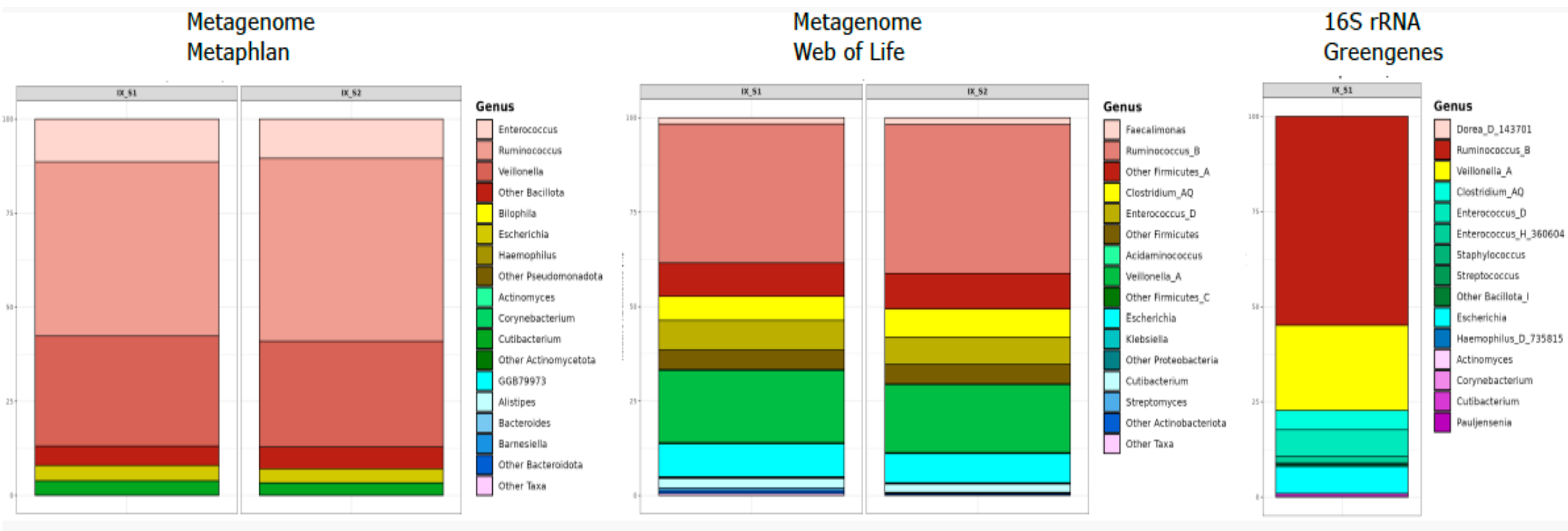

Supplementary Tables

Supplementary Table S1. Metadata and their definitions used in the PREVENT study including baseline and current characteristics. See Supplementary Table S1 xls.

Supplementary Table S2. Relative abundance in No-Bifidobacterium samples. The mean relative abundance of the 10 most abundant species in the No-*Bifidobacterium* samples compared to that in those containing *Bifidobacterium* spp. (top). Maximum relative abundance of 10 species that are highest in No-*Bifidobacterium* samples (bottom).

	**mean relative abundance**	
	**No-Bif**	**Bif**
Clostridium neonatale	24.3%	3.3%
Veillonela parvula	7.4%	2.1%
Escherichia coli	7.4%	2.3%
Phocaeicola vulgatus	6.3%	1.4%
Parabacteroides distasosis	3.1%	0.9%
Bacteroides fragilis	3.0%	3.1%
Clostridium butyricum	2.7%	0.3%
Thomasclavelia ramosum	2.8%	0.9%
Ruminococcus gnavus	2.6%	2.0%
Klebsiella michiganensis	2.2%	0.2%

	**maximum abundance**
	**No-Bif**
Enterobacter roggenkampii	69%
Citrobacter koseri	64%
Hungatella hathewayi	50%
Streptococcus vestibularis	40%
Enterocloster clostridioformis	32%
Akkermansia muciniphila	34%
Collinsella SGB47280	32%
Actinomyces urogenitalis	31%
Blautia faecis	27%
Sellimonas intestinalis	25%

Supplementary Table S3. MetaCyc predicted functions. Metagenome-based MetaCyc predicted functions in samples from infants containing gut *Bifidobacterium* spp (top) or no-*Bifidobacterium* (bottom). See Supplementary Table S3 xls.

Supplementary Table S4. KEGG predicted genes. Metagenome-based KEGG predicted genes in samples from infants containing gut *Bifidobacterium* spp (top) or no-*Bifidobacterium* (bottom). Discussed genes are indicated - see also Fig.4. See Supplementary Table S4 xls.
